# Distribution of Soil Extracellular Enzymatic, Microbial, and Biological Functions in the C and N-Cycle Pathways Along a Forest Altitudinal Gradient

**DOI:** 10.3389/fmicb.2021.660603

**Published:** 2021-09-03

**Authors:** Mohammad Bayranvand, Moslem Akbarinia, Gholamreza Salehi Jouzani, Javad Gharechahi, Petr Baldrian

**Affiliations:** ^1^Faculty of Natural Resources and Marine Sciences, Tarbiat Modares University, Tehran, Iran; ^2^Laboratory of Environmental Microbiology, Institute of Microbiology, Czech Academy of Sciences, Prague, Czechia; ^3^Microbial Biotechnology Department, Agricultural Biotechnology Research Institute of Iran, Agricultural Research, Education and Extension Organization, Karaj, Iran; ^4^Human Genetics Research Centre, Baqiyatallah University of Medical Sciences, Tehran, Iran

**Keywords:** forest soils, litter quality, enzyme activity, microbial entropy, N stock

## Abstract

The diverse chemical, biological, and microbial properties of litter and organic matter (OM) in forest soil along an altitudinal gradient are potentially important for nutrient cycling. In the present study, we sought to evaluate soil chemical, biological, microbial, and enzymatic characteristics at four altitude levels (0, 500, 1,000, and 1,500 m) in northern Iran to characterize nutrient cycling in forest soils. The results showed that carbon (C) and nitrogen (N) turnover changed with altitude along with microbial properties and enzyme activity. At the lowest altitude with mixed forest and no beech trees, the higher content of N in litter and soil, higher pH and microbial biomass nitrogen (MBN), and the greater activities of aminopeptidases affected soil N cycling. At elevations above 1,000 m, where beech is the dominant tree species, the higher activities of cellobiohydrolase, arylsulfatase, β-xylosidase, β-galactosidase, endoglucanase, endoxylanase, and manganese peroxidase (MnP) coincided with higher basal respiration (BR), substrate-induced respiration (SIR), and microbial biomass carbon (MBC) and thus favored conditions for microbial entropy and C turnover. The low N content and high C/N ratio at 500-m altitude were associated with the lowest microbial and enzyme activities. Our results support the view that the plain forest with mixed trees (without beech) had higher litter quality and soil fertility, while forest dominated by beech trees had the potential to store higher C and can potentially better mitigate global warming.

## Introduction

In terrestrial ecosystems, forest soils, as the largest carbon (C) reservoir, play a pivotal role in C cycling (Nottingham et al., 2020) and can potentially affect nitrogen (N) dynamics (Ndossi et al., 2020). Many studies have indicated that geography, soil, and litter properties (soil pH, nutrient content, and water content) and aboveground biotic factors (stand composition and diversity) are important predictors of soil C and N stocking (Delgado-Baquerizo et al., 2017; Walker et al., 2018). In addition, altitude has long been considered a potential driver of soil nutrient turnover through its influence on the biotic and abiotic components of forest ecosystems (Gebrewahid et al., 2018). Although altitude may not have a direct impact on soil organic matter (SOM) turnover, it can still influence this process indirectly by shaping soil, climatic, and vegetation conditions (Sierra and Causeret, 2018; Devi and Sherpa, 2019). Climatic conditions (i.e., decreased temperature and humidity) are heavily influenced by altitude, which in turn affects vegetation distribution/composition and alters the quality and quantity of litter/soil characteristics and C loss (Kotas et al., 2018; Quan et al., 2019). Recent studies have documented how changes in climatic conditions and forest canopy composition associated with altitude can affect the litter decomposition rate (Cardelli et al., 2019), soil microbial/enzyme activities (Feng et al., 2019; D’Alò et al., 2021), and nutrient storage in different ways (Bello et al., 2015).

As the main source of SOM, tree species composition and diversity along an altitudinal gradient may have a strong impact on C and N cycling (Gebrewahid et al., 2018; Sierra and Causeret, 2018). Despite the importance of soil nutrient cycling in the characterization of soil biogeochemistry under varying forest ecosystems (Bello et al., 2015; Frouz, 2018), the nature of soil nutrient reservoirs and their turnover in different regions are largely unknown. SOM decomposition involves both chemical and biological processes, including enzymatic catalysis (Sierra and Causeret, 2018; Nottingham et al., 2019), which generally decrease with increasing altitude, resulting in slower decomposition of organic matter (OM) (D’Alò et al., 2021).

Soil extracellular enzyme activities are essential for C and N cycling/stocking and litter decomposition and thus for microbial life (Baldrian and Štursová, 2011; Šnajdr et al., 2013; D’Alò et al., 2021). They also play an essential role in soil fertility as useful indicators for soil management (Baldrian et al., 2010; Cardelli et al., 2019). Changes in tree species composition along an altitudinal gradient alter soil microbial activity and thus the activity of soil enzymes (Feng et al., 2019; Ndossi et al., 2020). In fact, enzyme activity is sensitive to changes in vegetation and is closely related to soil primary quality, and health (Šnajdr et al., 2013; Nottingham et al., 2019). Soil nutrients (Cardelli et al., 2019), forest stands (Šnajdr et al., 2013), and climate (Walker et al., 2018) are factors affecting extracellular enzyme activity along altitudinal gradients. Recent studies have shown that the activity of C- and N-dependent enzymes has different trends with increasing altitude (Ndossi et al., 2020; D’Alò et al., 2021). The effects of altitude on soil extracellular enzyme activities are highly variable across individual studies (Lladó et al., 2017), since they do not follow simple biochemical rules (Baldrian et al., 2013). It is widely assumed that extracellular enzyme activity increases with increase in temperature, but in the forest altitudinal gradient their activity is more related to the quality of available OM, vegetation, and nutrient demands of the microbial biomass (Nottingham et al., 2019; D’Alò et al., 2021), which can have different trends with altitude (Lladó et al., 2017; Ndossi et al., 2020). Meng et al. (2020) reported that the higher C content of the soils at the higher altitude may denote a bigger labile C availability for C-dependent extracellular enzymes.

Moreover, knowledge about the function and activity of soil microorganisms along altitude is essential for understanding forest soil productivity (Massaccesi et al., 2020), as soil microbes play a fundamental role in biogeochemical cycling (Walker et al., 2018). Soil microbes play a pivotal role in the mineralization and decomposition of SOM by producing various hydrolytic enzymes (Cardelli et al., 2019). In particular, basal respiration (BR), substrate-induced respiration (SIR), microbial biomass C (MBC), microbial biomass N (MBN), and metabolic and microbial quotients as microbial indicators change with forest vegetation along the altitudinal gradient, while soil organic C (SOC) is often less affected (Walker et al., 2018; Massaccesi et al., 2020). In most cases, MBC and MBN showed different trends with changes of forest types along the altitudinal gradient (Massaccesi et al., 2020; Ndossi et al., 2020). Climatic conditions, vegetation, and edaphic factors also contribute to changes in substrate availabilities and microbial activities (Walker et al., 2018; Ndossi et al., 2020). Trees, as the primary source of plant biomass, affect microbial processes in ecosystems *via* specific litter chemistry and rhizodeposition (Šnajdr et al., 2013). Moreover, soil microbial activities are directly linked to C and N turnover/stock (Urbanová et al., 2015; Razanamalala et al., 2018) and mineralization of OM (Walker et al., 2018; Nottingham et al., 2019).

Soil fauna represents another important driver of SOM dynamics (García-Palacios et al., 2013; Frouz, 2018). Soil macro- and meso-organisms play key roles in nutrient cycling and SOM decomposition through their own metabolism, stimulation of microbial activity, and bioturbation (Frouz, 2018). Soil fauna is known as the engineer of the soil ecosystem that interacts with the soil microbiome and influences litter decomposition and nutrient cycling (García-Palacios et al., 2013). Climate directly alters litter decay because of the sensitivity of processes mediated by soil fauna to factors such as temperature or precipitation (García-Palacios et al., 2013). Recent studies have identified environmental factors (i.e., temperature and moisture) (García-Palacios et al., 2013), stand composition (Massaccesi et al., 2020), and soil and litter properties (Bayranvand et al., 2017b) as the main factors influencing the activity of soil fauna and flora in natural forests along altitudinal gradients (Bayranvand et al., 2021b).

Hyrcanian forests are located south of the Caspian Sea in northern Iran and are covered by temperate broadleaf forests (Bayranvand et al., 2017b), which are similar to vegetation in parts of Europe, northern Turkey, and the Caucasus (Talebi et al., 2013). Forest stand composition and climate change with altitude (Talebi et al., 2013), which in turn affect soil microbial and enzyme activities, and nutrient turnover (Gebrewahid et al., 2018; Banday et al., 2019). The aim of the present study was to investigate (i) how a shift in forest stand composition along an altitudinal gradient affects litter and soil chemical properties and soil C and N cycling; (ii) how biological (earthworm, nematode, protozoa, and fine-root biomass), microbial (BR, SIR, and microbial biomass C and N), and enzymatic activity patterns change along an elevation gradient; and (iii) which factors determine C and N stocks across transects. We hypothesized that (i) the C stock increases with altitude due to decreased litter decomposition and quality of beech forests and a low soil temperature (ST); (ii) the N stock is greater at low altitudes where tree diversity, pH, and litter quality are high; and (iii) the soil microbial respiration, MBC, and C-dependent enzymes are associated with higher soil and litter C with increasing altitude where beech forest are dominant, while the opposite is assumed for the N stock.

## Materials and Methods

### Study Area

**FIGURE 1:**
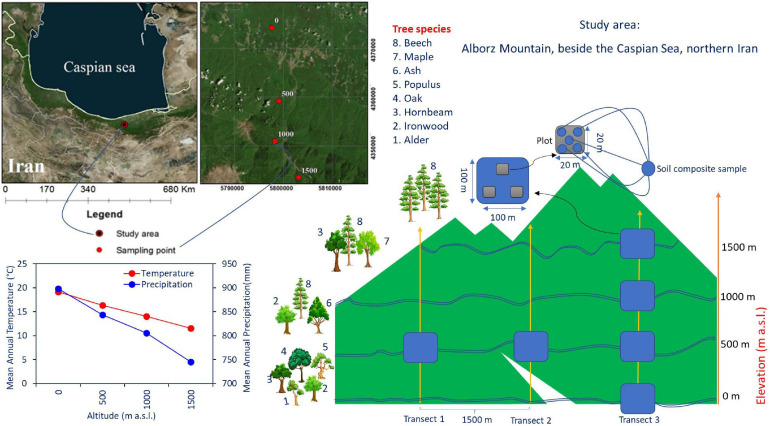
The study site at the Experimental Forest Station, Vaz watershed in the Central Caspian region of northern Iran, and tree species composition along the altitudinal gradient in the Alborz Mountains. The tree species growing in the area were *Fagus orientalis* Lipsky (beech), *Parrotia persica* C.A.M. (ironwood), *Carpinus betulus* L. (hornbeam), *Quercus castaneifolia* C.A.M. (oak), *Fraxinus excelsior* L. (ash), *Acer velutinum* (maple), *Alnus subcordata* C.A.M. (alder), and *Populus alba caspica* Bornm (Persian poplar).

Soil and litter samples were collected in the Hyrcanian mountain forest along three parallel altitudinal transects from 0 to 1,500 m a.s.l. in Mazandaran Province in northern Iran (57°90′–58°20′ E 43°30′–43°90′ N) (Figure 1). The region has a sub-Mediterranean climate where the mean annual temperature (MAT) decreases from 15 to 5°C (4–5°C decrease in temperature per 1,000 m), and the mean annual precipitation (MAP) decreases from 1,200 to 600 mm along an altitudinal gradient. Approximately 35–45% of the rainfall occurs in autumn, 18–35% in winter, and the rest (10–20%) in summer (Noushahr city meteorological station; for the period 1977–2010). The MAP is approximately 898, 843, 805, and 746 mm at 0, 500, 1,000, and 1,500 m a. s. l., respectively (Karger et al., 2017; Figure 1). The MATs at 0, 500, 1,000, and 1,500 m a. s. l. are 19.2, 16.3, 14, and 11.6°C, respectively. Based on clear changes in tree canopy composition along elevation, four altitude classes based on dominant trees were identified from 0 to 1,500 m a.s.l. (Khaleghi et al., 1997): including (1) plain mixed forests (0 m) with ironwood (*Parrotia persica* C.A.M.), oak (*Quercus castaneifolia* C.A.M.), hornbeam (*Carpinus betulus* L.), alder (*Alnus subcordata*), and Persian poplar (*Populus alba caspica* Bornm); (2) low mountainous mixed forests (500 m) with beech (*Fagus orientalis* Lipsky), ash (*Fraxinus excelsior* L), and ironwood; (3) middle mountainous mixed forests (1,000 m) with beech, maple (*Acer velutinum* Boiss), and hornbeam; and (4) high mountainous pure forests (1,500 m) with pure beech.

### Sample Collection and Laboratory Analysis

The study area constitutes an altitude gradient from 0 to 1,500 m along the Albourz Mountains on the southern coast of the Caspian Sea. At each elevation level, three 1-ha plots with a horizontal distance of 1,500 m were sampled. Each of these main plots was subdivided into three random subplots (each 400 m^2^). Samples were collected from corners and the center of each subplot (five samples per subplot, Figure 1). Elevation at each main plot was recorded using a Garmin model GPSMAP 60Cx. All living trees and the diameter of the trees at breast height (DBH, 1.3 m) were measured in each subplot with diameter tape for calculating of trees basal area (Table 1). Litter and soil samples were collected during May 2018. In total, 180 samples were collected for laboratory analyses (four altitude levels × three main plots × three subplots × five profiles). The soil and litter samples collected in each subplot were pooled separately and processed as an independent replicate for each elevation level.

**TABLE 1:** Distribution of forest types and properties along an altitudinal gradient in the Hyrcanian forest profile (Vaz catchment, Alborz Mountains, northern Iran).

Altitude (m a.s.l.)	Land cover (abbreviation)	Tree species density (number of trees/ha)	Tree species basal area (m^2^/ha)	Soil texture
1,500 ± 30	High mountainous pure forests (HMPF)	Beech (586.11)	Beech (17.49)	Clay loam (Clay = 34, Silt = 33, Sand = 33%)
1,000 ± 30	Middle mountainous mixed forests (MMMF)	Beech (91.67), hornbeam (36.11), maple (33.33)	Beech (28.41), maple (20.86), hornbeam (11.27)	Clay loam (Clay = 38.4, Silt = 29.2, Sand = 32.4%)
500 ± 30	Low mountainous mixed forests (LMMF)	Beech (203.56), ash (98.22), ironwood (75)	Ironwood (20.91), ash (11.63), beech (5.55)	Clay loam (Clay = 33.14, Silt = 32.86, Sand = 34%)
0 ± 30	Plain mixed forests (PMF)	Ironwood (194.44), oak (33.33), hornbeam (22.22), poplar (11.11), alder (2.28)	Ironwood (12.34), oak (11.24), poplar (5.90), hornbeam (2.59), alder (0.30)	Clay (Clay = 45.11, Silt = 34.44, Sand = 20.44%)

*The tree species growing in the area were (English name) Fagus orientalis Lipsky (beech), Parrotia persica C. A. Meyer (ironwood), Carpinus betulus L. (hornbeam), Quercus castaneifolia C.A.M. (oak), Fraxinus excelsior L. (ash), Acer velutinum (maple), Alnus subcordata C.A.M. (alder), and Populus alba caspica Bornm (Persian poplar).*

After digging the profile, organic layer thickness [i.e., OL, OF, and OH (in some profiles)] was measured with tape from the forest-floor surface to the top of the mineral soil (Bayranvand et al., 2021a). Subsequently, composite litter samples including OL and OF layers were obtained (Bayranvand et al., 2017a). After removing organic layers, soil samples were collected with plastic tubes (5 cm diameter). At the same time, earthworms were counted in a metal frame (30 × 30 × 10 cm) in both organic and mineral layers. ST was also recorded with a portable temperature probe (model: TA-288).

Litter samples were transported to the laboratory, dried at 65°C in a fan-assisted oven for 48 h and crushed/homogenized with an electric stamp for subsequent chemical analysis.

Soil samples were kept in a Styrofoam box filled with dry ice and transferred to the laboratory. Soil samples were sieved using a<2-mm mesh and either kept at 4°C for the measurement of chemical and soil respiration or freeze-dried and stored at −20°C for subsequent enzyme activity assays.

### Chemical, Biological, and Microbial Properties

The organic C content in litter samples was determined by dry combustion at 450°C for 4 h. The N content was measured using the micro-Kjeldahl technique (Bremner et al., 1982). Soil pH was measured with an Orion Analyser Model 901 pH meter in a 1:2.5 soil: deionized water slurry. Bulk density was calculated by the cold method [soil dry weight/core volume basis (Plaster, 1985)]. Soil moisture (SM) was measured by drying soil samples at 105°C for 24 h. SOC and total N (TN) were measured by the modified method of Walkley–Black (wet oxidation) (Allison, 1975) and micro-Kjeldahl techniques (Bremner et al., 1982). C and N contents were calculated by taking into account soil bulk density and depth (Plaster, 1985). MBC (Anderson and Domsch, 1990) and MBN (Brookes et al., 1985) were assessed by chloroform fumigation extraction using 5 g fresh soil and 20 ml 0.5 M K_2_SO_4_. MBC was calculated as the difference between organic C extracted from fumigated and non-fumigated soils using a conversion factor of 0.45. MBN was calculated by subtracting extracted N from fumigated and non-fumigated soils using a conversion factor of 0.54. Soil BR and SIR were measured by trapping CO_2_ emitted from fresh soil with NaOH in glass tubes during 5 days of incubation at 25°C (Alef, 1995) and titration with HCl. Glucose (1%) was used as a substrate for the SIR measurement. The ratio of the carbon availability index (CAI), metabolic quotient (qCO_2_), and microbial entropy ratio were calculated by dividing BR to SIR, BR to MBC, and MBC to C_*org*_, respectively (Cheng et al., 1996).

To demonstrate soil biological activity, earthworm, nematode and protozoan population densities as well as fine-root biomass were measured. Earthworms were enumerated in a 30-cm^2^ area and 10-cm depth (Bayranvand et al., 2017b). Nematode and protozoal densities were estimated in 100 g fresh soil by the modified cotton-wool filter method. After adding 1 g glucose and centrifugation, the numbers of nematodes (Liang et al., 2009) and protozoa (Mayzlish and Steinberger, 2004) were counted under a microscope. To determine the biomass of fine roots (<2 mm diameter), roots were extracted from 10-cm^3^ soil samples, washed with water, dried at 65°C, and weighed (Neatrour et al., 2005).

### Enzyme Activity Assays

In our study, the potential of exocleaving hydrolytic, endocleaving polysaccharide hydrolases, and ligninolytic enzyme activities were measured in laboratory. To assay exocleaving hydrolytic enzymes, 0.5 g of fresh soil was homogenized in 50 ml of 50 mM sodium acetate (pH 5.0) using an UltraTurrax (IKA Labortechnik, Germany) for 3 min at 8,000 rpm in an ice bath. The activities of arylsulfatase (EC 3.1.6.1), 1,4-α-glucosidase (EC 3.2.1.20), cellobiohydrolase (exocellulase; EC 3.2.1.91), 1,4-β-glucosidase (EC 3.2.1.21), 1,4-β-xylosidase (EC 3.2.1.37), N-acetylglucosaminidase (chitinase, EC 3.2.1.30), phosphomonoesterase (PME, EC 3.1.3.2), alanine aminopeptidase (EC 3.4.11.12), and leucine aminopeptidase (EC 3.4.11.1) were measured in homogenate using 4-methylumbelliferol- (MUF) or 7-amido-4-methylcoumarin- (AMC)-based substrates as described previously (Baldrian, 2009). The substrates were dissolved in DMSO at a final concentration of 500 μM. For background fluorescence subtraction, 200 μl of 50 mM sodium acetate (pH 5.0) was combined with 40 μl of MUF standards to correct the results for fluorescence quenching. The multiwell plates were incubated at 40°C, and the fluorescence was recorded after 5 min and until 125 min using a microplate reader (Infinite, TECAN, Austria) at an excitation wavelength of 355 nm and an emission wavelength of 460 nm. Enzymatic activities were calculated based on a standard curve of MUF or AMC. One unit of enzyme activity was defined as the amount of enzyme releasing 1 nmol of MUF or AMC per min and expressed per gram of soil dry mass.

To analyse the activity of endocleaving polysaccharide hydrolases and ligninolytic enzymes, homogenized samples were extracted at 4°C for 2 h on an orbital shaker (100 rpm) with 100 mM phosphate buffer, pH 7 (16: 1 w/v), filtered through Whatman #5 filter paper and desalted using PD-10 desalting columns (Pharmacia, Sweden) according to the supplier’s protocol to remove inhibitory small-molecular mass compounds (Baldrian, 2009). Laccase (EC 1.10.3.2) activity was measured by monitoring the oxidation of 2,2′-azinobis-3-ethylbenzothiazoline-6-sulfonic acid in citrate–phosphate (100 mM citrate, 200 mM phosphate) buffer, pH 5.0, at 420-nm wavelength. Manganese peroxidase (MnP, EC 1.11.1.13) was assayed in succinate–lactate buffer (100 mM, pH 4.5). 3-Methyl-2-benzothiazolinone hydrazone and 3,3-dimethylaminobenzoic acid were oxidatively coupled by the enzymes, and the resulting purple indamine dye was detected spectrophotometrically at 595 nm. The results were corrected using the activities of the samples without manganese (for MnP); the addition of manganese sulfate was substituted by an equimolar amount of ethylenediaminetetraacetic acid (EDTA). Endo-1,4-β-glucanase (endocellulase; EC 3.2.1.4) and endo-1,4-β-xylanase (EC 3.2.1.8) activities were measured with azo-dyed carbohydrate substrates (carboxymethyl cellulose and birch wood xylan, respectively) using the protocol of the supplier (Megazyme, Ireland). The reaction mixture contained 0.2 ml of 2% dyed substrate in 200 mM sodium acetate buffer (pH 5.0) and 0.2 ml of the sample. The reaction mixture was incubated at 40°C for 60 min and stopped by adding 1 ml of ethanol followed by vortexing for 10 s and centrifuging for 10 min at 10,000 × *g* (Baldrian, 2009). The amount of released dye was measured at 595 nm, and the enzyme activity was calculated according to standard curves correlating the dye release with the release of reducing sugars. One unit of enzyme activity was defined as the amount of enzyme releasing 1 mmol reducing sugars per minute. Enzyme activity was expressed per g soil dry mass.

### Statistical Analysis

To test for normality and equality of the variances of variables, Kolmogorov–Smirnov and Levene’s tests were employed, respectively. The differences in litter floor and soil physicochemical, biological, biochemical, and enzyme activities across the four forest types along an altitudinal gradient were analysed using one-way analysis of variance (one-way ANOVA). Means were compared using Tukey tests (HSD test). *P*-values < 0.05 were considered statistically significant. To examine associations between litter floor, soil data, and enzyme activities, a multivariate ANOVA analysis based on principal component analysis (PCA) was performed using the “ellipse” package in R version 3.3.2 (R Core Team, 2016). The two first axes with higher explained variations were plotted in PCA. Selected for further interpretation of the results, their relationship with all parameters was determined through a correlation matrix.

## Results

### Litter and Soil Chemical, Biological, and Microbial Properties

As expected, the altitude gradient greatly influenced tree canopy composition, litter quality, and quantity and soil chemical and biological properties, but with different trends (Tables 1, 2). Litter C was significantly elevated at 1,000–1,500-m altitudes, while litter N was significantly higher at the lowest altitude (0 m) than at the other altitude levels. In addition, the lowest litter C/N ratio and thickness were also found at this altitude level and increased with altitude (Table 2). The highest SM was recorded at 1,500 m. ST, however, sharply decreased with increasing altitude. The highest soil pH was recorded at the lowest (0 m) and highest (1,500 m) altitudes. Soil C was higher at elevations above 1,000 m, while soil N was significantly higher at the 0-m altitude (Table 2). The lowest soil C/N ratio was recorded at the 0-m altitude, and its content increased with altitude, particularly in beech forests (Table 2).

**TABLE 2:** Litter and soil chemical, biological, and microbial properties along an altitudinal gradient.

		Altitudinal gradient								Summary ANOVA	
Litter and soil properties		0 m		500 m		1,000 m		1,500 m		results	
		Mean	SE	Mean	SE	Mean	SE	Mean	SE	*F* test	*P-*value
Litter properties	C (%)	45.50 ab	1.65	44.06 b	1.47	48.99 a	0.46	49.74 a	0.40	5.677	0.003
	N (%)	1.49 a	0.06	0.99 c	0.05	1.34 ab	0.08	1.18 bc	0.05	12.454	<0.001
	C/N	30.73 b	1.19	44.93 a	1.94	37.98 ab	3.17	42.89 a	1.95	8.373	<0.001
	Thickness (cm)	2.40 c	0.33	1.01 c	0.24	4.33 b	0.56	9.84 a	0.56	76.370	<0.001
Soil physical and chemical properties	Moisture (%)	25.03 b	0.56	27.42 ab	0.76	26.10 b	1.72	31.24 a	1.73	4.298	0.012
	Temperature (°C)	17.59 a	0.22	17.82 a	0.15	13.81 b	0.12	10.59 c	0.14	436.04	<0.001
	Bulk density (g cm^–3^)	1.65	0.08	1.66	0.05	1.52	0.05	1.49	0.06	1.984	0.136
	pH (1:2.5 H_2_O)	7.84 a	0.01	6.45 b	0.14	6.09 b	0.17	7.56 a	0.08	51.872	<0.001
	C (%)	2.99 b	0.04	3.00 b	0.07	4.94 a	0.36	5.44 a	0.37	24.283	<0.001
	N (%)	0.74 a	0.03	0.48 b	0.03	0.56 ab	0.06	0.65 ab	0.08	4.311	0.012
	C/N	4.14 c	0.25	6.37 b	0.37	9.18 a	0.60	8.89 a	0.61	24.056	<0.001
Soil microbial and biological properties	MBC (mg kg^–1^)	1022.9 b	133.4	1309.2 b	25.81	6496.4 a	1012.7	1887.2 b	198.4	24.396	<0.001
	MBN (mg kg^–1^)	1134.2 a	87.61	303.78 b	86.95	173.6 b	54.98	154.7 b	15.3	47.078	<0.001
	Earthworm density (n m^–2^)	2.22 b	0.56	10.00 a	2.66	1.48 b	0.59	3.33 b	0.96	7.020	0.001
	Total nematode (in 100 g soil)	115.56 b	12.37	231.11 ab	31.82	204.44 a	42.92	124.44 b	15.56	4.085	0.015
	Protozoa density (×10^3^ g soil)	72.03 b	1.97	89.46 b	5.47	136.49 a	14.13	80.25 b	6.46	12.097	<0.001
	Fine root biomass (g m^2^)	17.71 b	4.93	26.76 b	9.49	48.27 ab	12.88	82.11 a	8.16	9.454	<0.001

*Different letters in each line indicate significant differences (P < 0.05 Tukey HSD test) between altitudes.*

**FIGURE 2:**
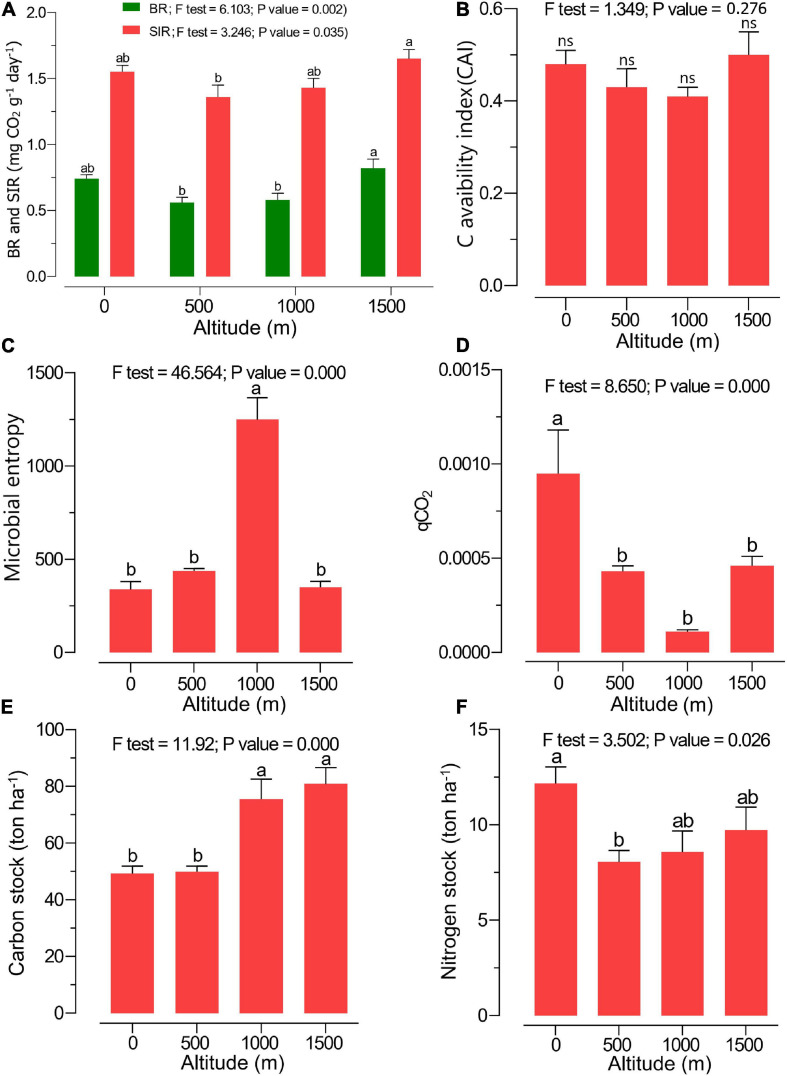
Soil microbial/biochemical properties such as **(A)** soil basal respiration (BR; green) and substrate-induced respiration (SIR; red), **(B)** carbon availability index (CAI), **(C)** microbial entropy ratio, **(D)** metabolic quotient (qCO_2_), **(E)** carbon stock, and **(F)** nitrogen stock at various altitudes. Different letters indicate a significant difference along the altitudinal gradient [analysis of variance (ANOVA) followed by Tukeys HSD *post hoc* test; *p* < 0.05].

The highest amount of MBC was observed at 1,000 m, while the highest MBN was found at the 0-m altitude level, and the content decreased along the elevation gradient (Table 2). A significantly higher soil BR and SIR were observed at 1,500 m in pure beech forests compared with 0 > 500 ≈ 1,000 m altitude levels (Figure 2A). The CAI ratio did not change along the altitudinal gradient (Figure 2B). Microbial entropy was enhanced at the 1,000-m altitude compared with other altitude levels (Figure 2C), while the lowest altitude (0 m; mixed plain forest) showed the highest qCO_2_ ratio compared with other levels (mixed and pure beech forests) (Figure 2D). The soil C stock was highest under mixed and pure beech forests at 1,000- to 1,500-m elevation levels compared with the lower altitudes (0–500 m) (Figure 2E). The N stock was significantly higher at 0-m altitude under plain forest than under pure forest (1,500 m) (Figure 2F). The highest density of earthworms was found at 500 m, whereas the total protozoan density was highest at 1,000 m (Table 2). Fine-root biomass also increased along the altitudinal gradient (Table 2).

### Soil Enzyme Activity

**FIGURE 3:**
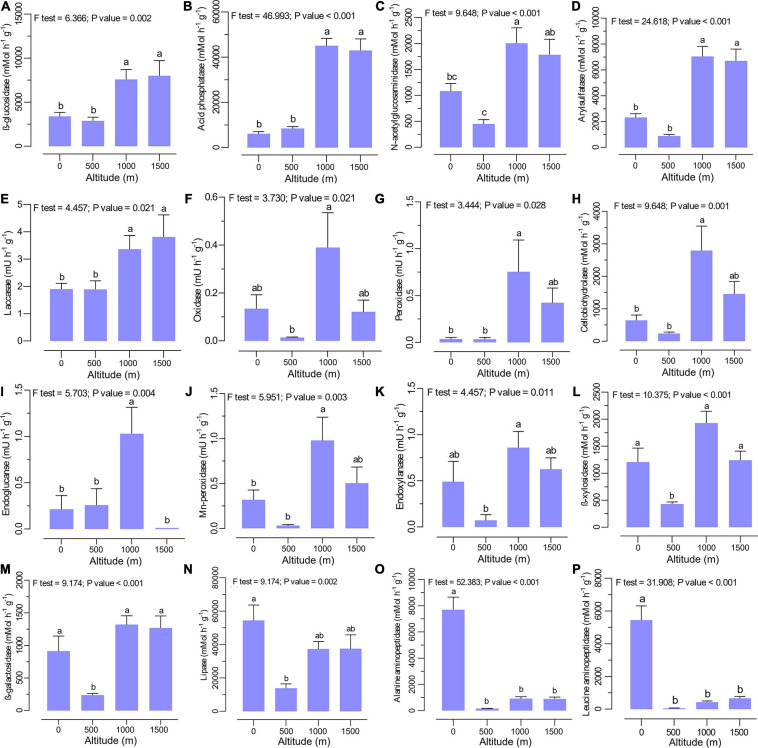
Soil extracellular enzyme activities such as **(A)** β-glucosidase, **(B)** acid phosphatase, **(C)** N-acetylglucosaminidase, **(D)** arylsulfatase, **(E)** laccase, **(F)** oxidase, **(G)** peroxidase, **(H)** cellobiohydrolase, **(I)** endoglucanase, **(J)** Mn-peroxidase, **(K)** endoxylanase, **(L)** β-xylosidase, **(M)** β-galactosidase, **(N)** lipase, **(O)** alanine aminopeptidase, and (P) leucine aminopeptidase at various altitudes. Different letters indicate a significant difference along the altitudinal gradient (ANOVA and Tukey HSD test; *p* < 0.05).

The extracellular enzyme activities were significantly different between altitudes with no clear trend (Figures 3A–P). The activities of β-glucosidase (Figure 3A), acid phosphatase (Figure 3B), N-acetylglucosaminidase (Figure 3C), arylsulfatase (Figure 3D), and laccase (Figure 3E) were significantly higher at higher elevation levels (1,000 to 1,500 m). Specifically, higher activities of cellobiohydrolase (Figure 3H), endoglucanase (Figure 3I), endoxylanase (Figure 3K), oxidase (Figure 3F), peroxidase (Figure 3G), and Mn-peroxidase (Figure 3J) were observed at the middle altitude level (1,000 m), where mixed beech forest is dominant. In contrast, the activities of lipase (Figure 3N), alanine aminopeptidase (Figure 3O), and leucine aminopeptidase (Figure 3P) declined with increasing altitude, with the highest activities in plain forests (0 m altitude level). The lowest activity for β-xylosidase (Figure 3L) and β-galactosidase (Figure 3M) was recorded at 500 m with no clear trend across the altitudinal gradient.

### The Relationship Among All Litter and Soil Characteristics

**FIGURE 4:**
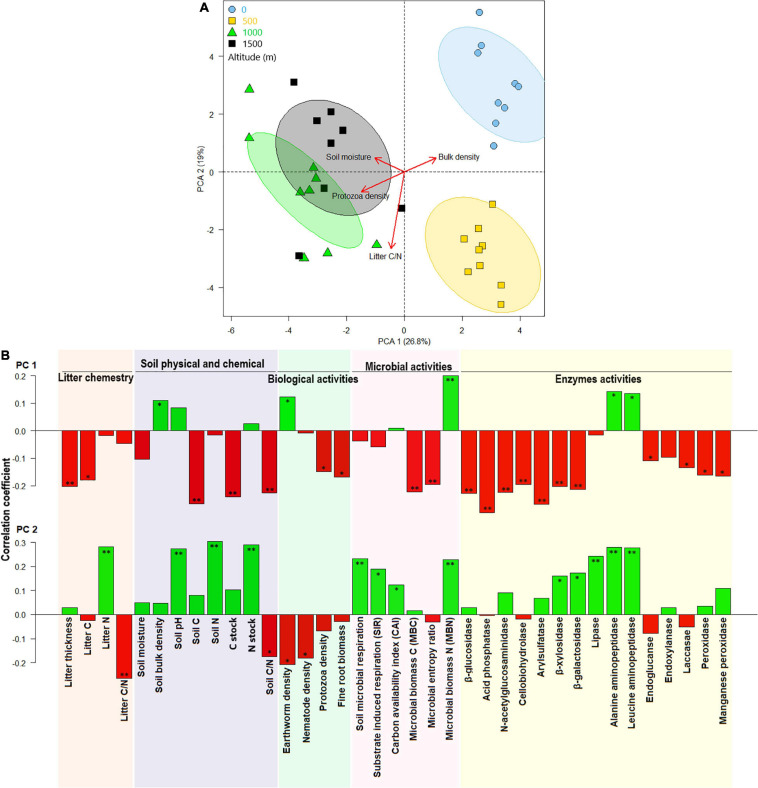
Principal component analysis (PCA) of chemical litter and soil properties and soil biological, biochemical, microbial, and enzyme activities at various altitudes **(A)** and their correlations with the first two canonical axes **(B)**. * and ** represents correlation coefficients significant level at *p* < 0.05 and *p* < 0.01, respectively.

Considering all litter and soil biological and biochemical properties and enzyme activities, samples were clustered separately by altitude in the PCA plot (Figures 4A,B). The two first axes of PCA covered ∼46% of the variation among samples collected from different altitudes. At the higher elevation (1,500 m), higher litter C, litter thickness, soil C, soil C/N ratio, protozoan density, fine-root biomass, MBC, and microbial entropy were associated with increased C stock and higher activities of β-glucosidase, acid phosphatase, N-acetylglucosaminidase, cellobiohydrolase, arylsulfatase, β-xylosidase, β-galactosidase, endoxylanase, peroxidase, and Mn-peroxidase enzymes (Figures 4A,B). Litter N, soil N, pH, BR, SIR, CAI, and MBN were aggregated at the positive portion of PC2 and were associated with an increased N stock and higher activities of lipase, alanine aminopeptidase, leucine aminopeptidase, β-xylosidase, and β-galactosidase enzymes at the 0-m-altitude level.

## Discussion

The tree composition of forests covering the northern part of the Alborz Mountains changes with altitude (Talebi et al., 2013), a condition that is associated with altered soil C and nutrient stocks. The high C stocks at high altitudes can be due to the continuous accumulation of leaf litter and slower decomposition because of the lower temperatures at the higher elevation levels (Banday et al., 2019). The dominance of *F. orientalis* Lipsky at high elevations may likely contribute to C sequestration, resulting in a higher litter C/N ratio and higher soil C compared with other deciduous forest stands at lower altitudes (Talebi et al., 2013; Kooch and Bayranvand, 2017). Compared with other tree species found at low altitudes, the beech forest floor is more recalcitrant to decomposition, which could be due to its lower nutrient contents (Delgado-Baquerizo et al., 2017), higher C/N ratio (Bayranvand et al., 2017a), and high lignin content (Kooch and Bayranvand, 2017). The differences in soil C stocks in the two forest types thus originate from their different vegetation types, as the quantity, and quality of litter are crucial drivers that affect the soil microbial biomass and soil pH (Banday et al., 2019; Massaccesi et al., 2020). Low temperatures at higher altitudes are another factor contributing to the higher C stock, as in cooler climates, soils have higher OM due to a slow mineralization rate and a high forest-floor thickness (Gebrewahid et al., 2018; Banday et al., 2019). We found that plain mixed forest with species of *P. persica*, *Q. castaneifolia*, and *C. betulus* at low altitudes had higher litter quality (high N and low C/N), higher soil fertilities and N stocks than beech forest stands at higher altitudes. Krashevska et al. (2017) noted that a high initial litter N concentration and low C/N ratio as well as low lignin and high holo-cellulose concentrations are associated with high litter quality and promote rapid decomposition (Šnajdr et al., 2013).

Previous analyses by Šnajdr et al. (2013) and Feng et al. (2019) suggested that tree species determine the quality and quantity of resources available for soil microorganisms and enzyme functions. Our analyses revealed that higher enzyme activities are typical of higher altitudes under beech forests with high litter thickness, litter, and soil C and C/N ratios under appropriate SM. Šnajdr et al. (2008) studied the relationship between enzyme production and litter decomposition by fungal communities under different forest covers and noted that the decomposition of litter components, such as cellulose and lignin, is often attributed to cellulase enzyme activities. In agreement, enzymes, such as laccase, endoglucanase, β-glucosidase, and β-xylosidase, were reported to be produced in *Fagus sylvatica* forest by *Lepista nuda* fungi. Mn-peroxidase and laccase contribute to lignin degradation, which results in both depolymerization and partial mineralization (Valášková et al., 2007). In the present study, the activities of β-glucosidase, acid phosphatase, N-acetylglucosaminidase, arylsulfatase, and laccase were higher at higher elevations (1,000 to 1,500 m) in beech forests where litter thickness, C and C/N ratios, fine-root biomass, and SM were significantly higher (Banday et al., 2019). Increased C-dependent enzyme activity is likely due to a higher availability of aboveground litterfall (with higher C) and fine-root necromass to soil microorganisms (Feng et al., 2019). In addition to a lower decomposition rate, litter quality also decreased with altitude (Marian et al., 2019).

Increased soil enzyme activities could be associated with higher C accumulation since soil microbial biomass and heterotrophic respiration increase with plant density (Feng et al., 2019). Kang et al. (2009) also noted that higher activities of acid phosphatase and oxidase were dependent on the soil C content. In previous studies, it has been reported that the activities of β-glucosidase, cellobiohydrolase, acid phosphatase, and N-acetylglucosaminidase positively correlate with soil C (Štursová and Baldrian, 2011; Xu et al., 2020). Our findings are consistent with recent studies highlighting that higher enzyme activities are positively correlated with altitude and C, particularly under beech forests with high C sources and MBC (Štursová and Baldrian, 2011; Šnajdr et al., 2013; Xiao et al., 2019).

Principal component analysis showed that the higher soil C, moisture, SIR, BR, MBC, and litter thickness at the highest altitude (1,500 m; under beech forests) were associated with C stock, β-glucosidase, N-acetylglucosaminidase, arylsulfatase, β-xylosidase, β-galactosidase, endoxylanase, peroxidase, and Mn-peroxidase enzyme activities. Similarly, Cardelli et al. (2019) showed a positive correlation between C and the β-glucosidase, cellulase, xylosidase, and glucuronidase activities under *F. sylvatica*. These enzymes are involved in the degradation of cellulose, pectin and lignin, which are the main components of forest-floor plant residues (Cardelli et al., 2019). Previous studies have also reported a correlation of arylsulfatase and acid phosphatase enzyme activities with soil C content in deciduous forests (Baldrian et al., 2013; Kooch and Bayranvand, 2017). We also showed the highest enzyme and biological activities (i.e., nematode and protozoa density) under mixed beech–hornbeam–maple forest with high MBC and moderate litter/soil qualities and thickness. This could be due to a high C in beech litter/soil and a high litter decomposition of hornbeam and maple trees (Kooch and Bayranvand, 2019). Dehydrogenases and oxidases facilitate the degradation of lignin and thus improve C cycling through the release of nutrients from plant residues (Massaccesi et al., 2020). Enzymes such as urease and alanine aminopeptidase are involved in N cycling in terrestrial ecosystems (Šnajdr et al., 2013; Feng et al., 2019).

In this study, the activities of alanine and leucine aminopeptidase were significantly higher in the plain mixed forest with high litter/soil N and pH, while their activities declined at high altitudes and at low temperatures. pH and N are both important factors affecting enzyme activity (Šnajdr et al., 2013). A general decline in qCO_2_ and N with altitude could be attributed to a decreased ST and pH (Massaccesi et al., 2020). Štursová and Baldrian (2011) showed that the specific activity of leucine aminopeptidase increases with pH and N. A decrease in pH at high altitudes likely results from leaching of bases from the surface due to high precipitation or higher concentrations of H^+^ ions due to higher litter decomposition (Banday et al., 2019). The PCA showed that the soil N stock was associated with higher litter N, pH, qCO_2_, MBN, and activities of lipase, alanine and leucine aminopeptidase enzymes. We also noted a clear association of chitinase and leucine-aminopeptidase enzymes with high soil quality (high N and low C/N), consistent with their activities under high fresh OM (Cardelli et al., 2019).

Soil C accumulation is enhanced by low ST and acidic and anaerobic conditions, all of which inhibit the decomposition process (Banday et al., 2019; Devi and Sherpa, 2019). As an important driver of the microbial community and microbial C limitation (Walker et al., 2018), ST was significantly lower at high altitudes than at low altitudes (Cui et al., 2019). Increased soil C and MBC are known to positively correlate with high SM and low ST (Baldrian et al., 2010; Quan et al., 2019).

The C stock was positively correlated with SM, MBC, BR, and SIR but negatively correlated with higher levels of ST. Microbial entropy and MBC were enhanced at middle altitudes (1,000 m), where the dominant tree species were beech, hornbeam, and maple. The activities of soil organisms, including earthworms, protozoa, and nematodes, depend on soil conditions, such as pH, N, and available nutrients, which are affected by the rate of OM decomposition (Kooch and Bayranvand, 2017). Soil N and pH are the main factors affecting litter decomposition, which is the major source of the soil microbial qCO_2_ under high temperatures (Quan et al., 2019). Hornbeam forest had the highest litter quality, soil fertility, and soil organism activities compared with beech forest. In fact, soils under hornbeam had higher nutrient concentrations and pH that make conditions more favorable for microbial activities, whereas poor beech litter quality was due to a lower N concentration, higher C/N ratio, and higher levels of recalcitrant compounds associated with lower MBN and N sequestration (Kooch and Bayranvand, 2017). Among chemical variables, N had the strongest effect on the microbial biomass in litter and soil (Urbanová et al., 2015). Thus, the higher rate of N mineralization under hornbeam can be linked to the lower C/N ratio of its topsoil (Kooch and Bayranvand, 2019).

## Conclusion

Microbial and enzyme activities can be used as sensitive indicators of soil function in response to different products of forest aboveground along altitudinal gradient, which helps to better understand the dynamics of both C and N cycling. Our findings indicate that N stock increases under plain mixed forest, which likely results from increased ST, tree diversity, litter N, MBN, pH, and leucine/alanine aminopeptidase enzymes. At higher altitudes, higher litter thickness, litter/soil C and C/N ratios, BR, SIR, and MBC and β-glucosidase, acid phosphatase, arylsulfatase, and laccase activities were associated with the lowest ST, highest SM, and thus greater C accumulation. In addition, our results showed that decreased litter/soil N and pH were associated with lower soil microbial and enzyme functions, which can be seen well at the 500-m altitude. Plain forests with high tree diversity, which produce high litter quality, had the highest N-dependent enzyme activity and soil fertilities, while mountainous forests with dominant beech trees had the potential to store more C and thus help to increase MBC. This study provides novel insights into different enzyme activities along altitude and improves our understanding of soil C and N cycling in forests.

## Data Availability Statement

The original contributions presented in the study are included in the article/supplementary material, further inquiries can be directed to the corresponding authors.

## Author Contributions

MA, GSJ, and JG are supervisors and advisors of the Ph.D. thesis of MB, respectively, and actively contributed to the design of the study. MB and PB performed the material preparation, data collection, and analysis. MB, PB, and MA analyzed and interpreted the soil properties, and enzyme activity data. MA contributed to identifying and classifying the vegetation cover. MB and JG analyzed the data. MB, PB, JG, and GSJ wrote the manuscript. All the authors contributed to the study conception and design, and read and approved the final manuscript.

## Conflict of Interest

The authors declare that the research was conducted in the absence of any commercial or financial relationships that could be construed as a potential conflict of interest.

## Publisher’s Note

All claims expressed in this article are solely those of the authors and do not necessarily represent those of their affiliated organizations, or those of the publisher, the editors and the reviewers. Any product that may be evaluated in this article, or claim that may be made by its manufacturer, is not guaranteed or endorsed by the publisher.
